# Celecoxib Suppresses the Phosphorylation of STAT3 Protein and Can Enhance the Radiosensitivity of Medulloblastoma-Derived Cancer Stem-Like Cells

**DOI:** 10.3390/ijms150611013

**Published:** 2014-06-18

**Authors:** Meng-Yin Yang, Hsu-Tung Lee, Chien-Min Chen, Chiung-Chyi Shen, Hsin-I Ma

**Affiliations:** 1 Department of Neurological Surgery, Jan-Ai General Hospital, Taichung 412, Taiwan; E-Mail: yangmy04@gmail.com; 2 Graduate Institute of Medical Sciences, National Defense Medical Center, Taipei 114, Taiwan; E-Mail: sdlee@vghtc.gov.tw; 3 Department of Neurological Surgery, Taichung Veterans General Hospital, Taichung 40705, Taiwan; 4 Department of Physical Therapy, Hungkuang University, Taichung 433, Taiwan; 5 Division of Neurological Surgery, Department of Surgery, Changhua Christian Hospital, Changhua 505, Taiwan; E-Mail: 96015@cch.org.tw; 6 Department of Neurological Surgery, Tri-Service General Hospital, National Defense Medical Center, Taipei 114, Taiwan; 7 Department of Medicine, National Defense Medical Center, Taipei 114, Taiwan

**Keywords:** medulloblastoma, STAT3, celecoxib, CD133, Nestin, cancer stem-like cells

## Abstract

Medulloblastoma (MB) is a malignant primary brain tumor with poor prognosis. MB-derived CD133/Nestin double-positive cells (MB-DPs) exhibit cancer stem-like cell (CSC)-like properties that may contribute to chemoradioresistance, tumorigenesis and recurrence. In various tumors, signal transducer and activator of transcription 3 (STAT3) upregulation including MB which can regulate the expression of Nestin. Celecoxib, a selective COX-2 inhibitor, has been shown to potentially reduce STAT3 phosphorylation. The aim of the present study was to investigate the role of celecoxib in enhancing the effects of ionizing radiotherapy (IR) on MB-DP. MB-DPs and MB-derived CD133/Nestin double-negative cells (MB-DNs) were isolated from medulloblastoma cell line Daoy. Then, both of them were treated with celecoxib in different concentrations, and cell viability was assessed. The assays of cell survival, sphere formation, radiosensitivity, colony formation, apoptotic activity and mouse xenografting experiments in MB-DPs and MB-DNs treated with celecoxib alone, radiation alone, or celecoxib combined with radiation were further evaluated. We isolated MB-DPs from MB cell line Daoy, which exhibited typical CSC-like characteristics. Microarray analysis and Western blotting both indicated the upregulation of Janus kinase (JAK)-STAT cascade and STAT3 phosphorylation. Incubation with celecoxib dose-dependently suppressed the CSC-like properties and enhanced the IR effect on the induction of apoptosis, as detected by TUNEL assay and staining for Caspase 3 and Annexin V. Finally, celecoxib also enhanced the IR effect to suppress tumorigenesis and synergistically improve the recipient survival in orthotopic MB-derived CD133/Nestin double-positive cells (MB-DP cells) bearing mice.

## 1. Introduction

Medulloblastoma (MB), a highly malignant primary brain tumor, comprises 13%–20% of all childhood brain tumors which is the most common pediatric brain tumor [1]. In the early literature, researchers have described that MB invades the embryonic posterior fossa of the cerebellum and this is believed to arise from the precursor cells of the external granule layer or neuroepithelial cells from the cerebellar ventricular zone of the developing cerebellum. Recent studies described that MBs can divide as different diseases originating from different locations within the cerebellum depending on molecular subgroup [2,3,4,5,6]. If patients do not receive active treatment, these patients are inclined to have recurrence and die within 3 years [7,8]. Since surgical excision alone is usually ineffective and may provide a poor overall survival for infants and young children, the standard treatment for of MB consists of aggressive surgery, craniospinal radiotherapy and chemotherapy [9]. The precise mechanisms underlying the pathogenesis of MB are still unclear, and the development of effective therapeutic strategies for MB is undoubtedly urgent.

In some recent studies, it has been suggested that a subset of cancer cells, known as cancer stem-like cells (CSCs), are the most aggressive cell type with high self-renewal and stemness properties in many malignant tumors [10,11]. These CSCs are responsible for cancer progression, metastasis and recurrence. CSC showed abundant expression of specific surface markers. CSCs can be identified and isolated by different methodologies, including sorting of CSCs by flow cytometry based upon cell surface marker expression and Hoechst side population analysis [12,13,14]. Among these markers, CD133 was demonstrated as a specific cell surface marker expressed on normal human neural stem cells (NSCs) and malignancies including colon, lung, and prostate cancers [11,15,16,17,18]. Some studies also suggested that CD133 expression in brain tumors could be a prognostic indicator for tumor recurrence, malignant progression, and patient prognosis [11,17,18]. In addition, the neural progenitor marker Nestin, is an intermediate filament (IF) protein, also abundantly produced in the developing central nervous system [19,20,21]. Similar to CD133, Nestin also plays a role in neurogenesis and was widely employed as a marker of neural stem cells in brain development [11,22].

Signal transducer and activator of transcription 3 (STAT3) is a transcription factor in the family of DNA-binding molecules that responds to cytokine and growth factor signaling. In addition, STAT3 also serves as a potential target for anticancer treatment and cancer prevention [23,24]. The phosphorylated STAT3 is constitutively activated or overexpressed in a variety of human cancers including brain tumors [25,26]. Remarkably, several lines of evidence have demonstrated that constitutively activated STAT3 serves a crucial role in MB tumorigenesis by controlling the expression of target genes, which can protect apoptosis and enhance cell proliferation [27]. Some articles showed that the JAK-STAT signaling pathway can upregulate the expression level of Nestin during central nervous system development [28,29]. Non-steroidal anti-inflammatory drugs (NSAIDs) are medications used to alleviate pain and to ameliorate inflammation. Of particular importance is that several investigations have demonstrated that NSAIDs also reduced tumor growth and have enhanced sensitivity to radiotherapy and chemoradiotherapy in mice. Celecoxib was the first cyclooxygenase-2 (COX-2)-selective NSAID which was demonstrated to possess potent anticancer activities against various human cancers [30,31]. The observations by Liu *et al.* indicated celecoxib as a novel STAT3 inhibitor. In addition, the anticancer effects by celecoxib have been demonstrated to be exerted through suppressing STAT3 phosphorylation [32]. However, the precise molecular mechanisms underlying the tumor-suppressing effect of celecoxib are still need to be investigated.

In the present study, we first attempted to isolate MB-derived CD133/Nestin double-positive cells (MB-DPs) from MB cell lines, Daoy and evaluate whether this subset of isolated cells exhibiting CSC-like characteristics exhibited typical CSC-like characteristics. We next assessed the treatment efficacy of celecoxib on STAT3 signaling, the CSC-like signature, MB tumorigenesis and the resistance of MB-DPs to radiotherapy. In *in vivo* studies, we also examined whether celecoxib treatment reduced tumorignenesis, improved recipient survival and sensitized the efficacy of radiotherapy in orthotopic MB-DP cells bearing mice.

## 2. Results and Discussion

### 2.1. Characterization of Cancer Stem-Like Properties in MB-CD133+ and Nestin+ Cell

**Figure 1:**
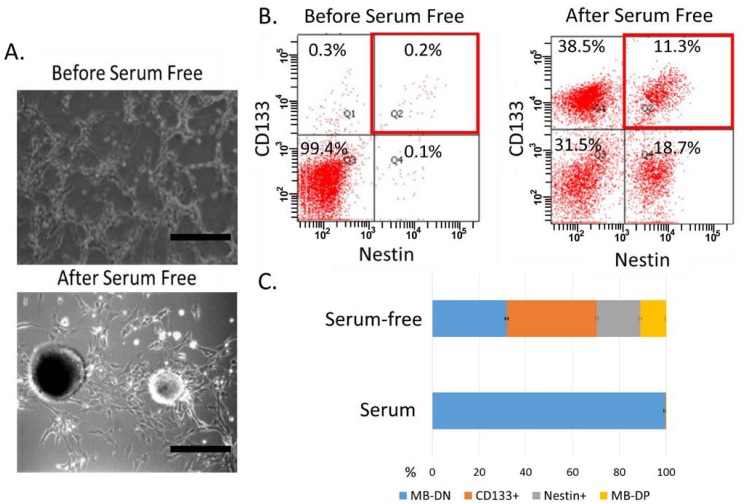
Enrichment of medulloblastoma-derived cancer cells double-positive cells for both CD133/Nestin (MB-DPs). (**A**) Daoy cells were cultured in the DF-12 serum-free medium with bFGF and EGF (20 ng/mL) for 4 weeks. Spheroid-like bodies were identified using phase contrast microscopy. Bar 100 μm; (**B**) Using fluorescence activated cell sorting, MB-DP cells were sorted from medulloblastoma cell line, Daoy. The populations of MB-DP cell populations were confirmed by flow cytometry; and (**C**) The percentages of the various sub-populations in Figure 1B. The experiments were repeated at least three times.

Previous evidences have demonstrated that cancer stem cells can be cultured and enriched without affecting their self-renewal capabilities in serum-free media with basic fibroblast growth factor (bFGF) and epidermal growth factor (EGF) [33,34,35]. In the present study, we also employ such serum-free culture condition to expand cancer stem-like cells in medulloblastoma cell lines, Daoy. We cultured and maintained these parental cells (Figure 1A, upper, representative morphology indicates Daoy) in serum-free media supplemented with bFGF and EGF for 1 month, and these cancer cells stably proliferated and formed floating spheroid-like bodies (SBs) (Figure 1A, lower, representative morphology indicates Daoy). As shown by the results of fluorescence activated cell sorting (FACS), our data indicated that the amount of medulloblastoma SB-derived cancer cells positive for CD133 were largely enriched by such conditions (Figure 1B,C). Similar data indicating enriched population of SB-derived cancer cells were detected using antibodies against another neural stem cell marker, Nestin (Figure 1B,C). We next employed the magnetic bead method to isolate medulloblastoma SB-derived cancer cells simultaneously positive for CD133 and Nestin (medulloblastoma-derived cancer cells double-positive cells for both CD133/Nestin, MB-DPs) and examined the cancer stem-like characteristics of MB-DPs in subsequent experiments.

### 2.2. Cancer Stem-Like Properties of MB-DPs

**Figure 2:**
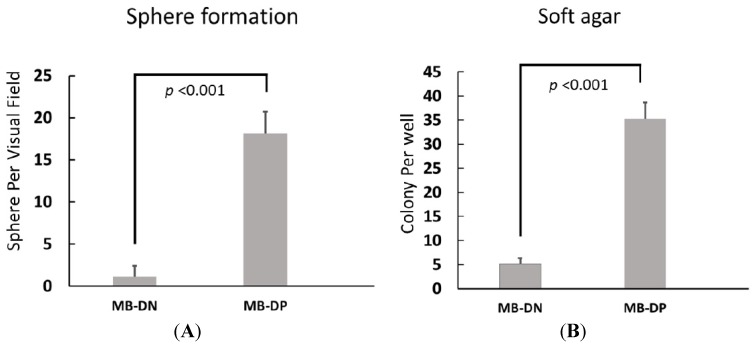
Formation of sphere-like bodies and soft agar colonies by MB-DPs. MB-DPs and MB-DNs from medulloblastoma cell lines Daoy was subjected to sphere formation assays and soft agar colony formation assay. (**A**) Comparison of sphere formation ability between MB-DPs and MB-DNs; and (**B**) Comparison of soft agar colony formation between MB-DPs and MB-DNs: MB-derived CD133/Nestin double-positive cells (MB-DPs); MB-derived CD133/Nestin double-negative cells (MB-DNs).

To further delineate the cancer stem-like properties and evaluate the *in vitro* tumorigenicity in these MB-DPs, we conducted sphere formation assay and soft agar colony assay. As shown in Figure 2A, abilities for both sphere formation and the formation of soft agar colonies were largely increased in MB-DPs from Daoy, compared with MB-derived CD133/Nestin double-negative cells (MB-DNs) or parental cells from corresponding medulloblastoma cell line (Figure 2, *p* < 0.001 *vs.* MB-DNs in both assays; MB-DPs *vs.* parental cells data not shown). Taken together, these findings indicated that MB-DPs isolated from medulloblastoma cell line exhibit typical cancer stem-like characteristics.

### 2.3. Upregulation of STAT-Related Pathways in MB-DP Cells

**Figure 3:**
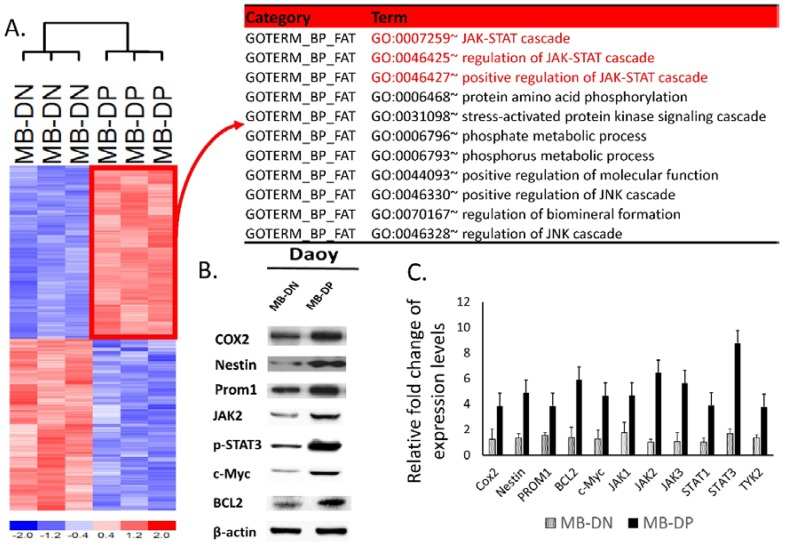
Differential gene expression between MB-DP and MB-DN cells. (**A**) Heatmap of differential gene expression between MB-DP and MB-DN cells. Gene expression microarray analysis showing genes that were differentially expressed in MB-DP and MB-DN cells. In bioinformatics analysis of differential gene expression, the JAK-STAT cascade and related pathway were found to be the most significantly different between MB-DP and MB-DN cells; (**B**) The results of western blot show the upregulation of Cox2, Nestin, CD133, phosphorylated-STAT3 and STAT3-related protein, JAK2, BCL2, c-Myc in MB-DP cells but not in MB-DNs; and (**C**) The data of real-time quantitative PCR show that the Cox2, Nestin, CD133 and STAT3-related genes were highly expression in MB-DPs but not in MB-DNs.

Bioinformatics provides a global vision to study the biological questions, especially for the analysis of microarray data. Results of microarray showed that the expression levels of STAT3 and STAT3-related pathways were consistently upregulated in MB-DPs as compared with MB-DNs (Figure 3A). Along with the findings of bioinformatics analysis and transcriptional profiling indicating the crucial roles of STAT3 and STAT3-related pathways in the regulation of stemness and tumorigenicity in MB-DP cells, we further examined the protein expression patterns by western blot and mRNA expression level by real-time quantitative PCR confirmed the upregulation of phosphorylated STAT3 and STAT3-related gene in MB-DPs both in protein and mRNA levels (Figure 3B,C). In contrast, the expression levels of STAT3, JAK2, BCL2 and c-Myc were low in MB-DNs (Figure 3B,C).

### 2.4. Celecoxib Suppressed STAT-Related Pathways in MB-DP Cells and Drove MB-DP Cells to Lose Their Cancer Stem-Like Gene Signatures

**Figure 4:**
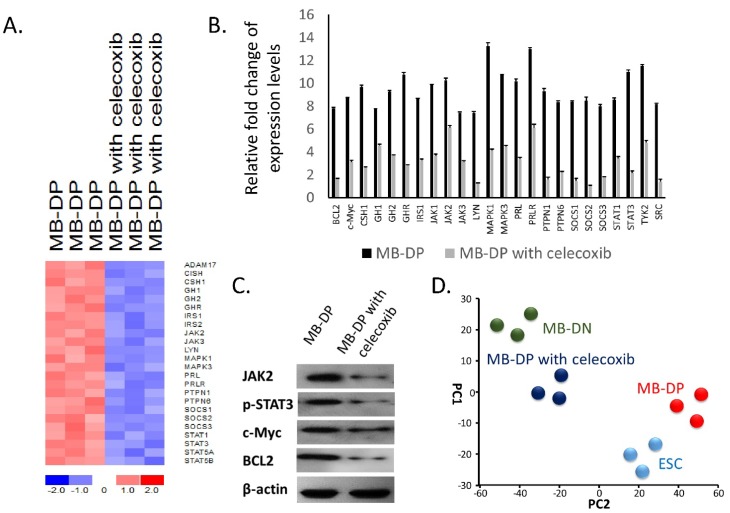
Celecoxib treatment shifted the gene signature profiles of MB-DPs towards that of MB-DN cells. (**A**) Heatmap of differential gene expression analysis showing STAT3-JAK3-related genes in MB-DP cells following treatment with celecoxib. STAT3-JAK3-related pathway gene expression levels were altered following treatment with celecoxib. STAT family genes and JAK1 gene were significantly downregulated by such treatment; (**B**) The data of real-time quantitative PCR show that the STAT3-related genes were highly suppressed in celecoxib treated MB-DPs than control MB-DPs; (**C**) Western blot showing the downregulation of phosphorylated-STAT3 and STAT3-related protein, JAK2, BCL2, c-Myc in celecoxib treated MB-DPs than control MB-DPs; and (**D**) Principle component analysis (PCA) illustrating the distances among MB-DP cells, celecoxib-treated MB-DP cells, MB-DN cells and embryonic stem cells (ESCs). The PC1 and PC2 axes are statistical units used in information visualization revealing the similarities and dissimilarities between the subjects.

Given that our data indicated a crucial role of STAT3 and STAT3-related pathways in the regulation of cancer stem-like properties in MB-DPs, we explored the gene signature profile of MB-DP cells incubated with or without 30 μM celecoxib. A hierarchical heatmap of MB-DP cells treated with or without celecoxib was generated based upon the results of gene expression microarray and bioinformatics analyses (Figure 4A). This heatmap and the data of real-time quantitative PCR indicated that the expression levels of STAT3-related gene were significantly downregualted by celecoxib treatment in MB-DPs (Figure 4A,B). The protein expression patterns by western blot showing the downregulation of phosphorylated-STAT3 and STAT3-related protein, JAK2, BCL2, c-Myc in celecoxib treated MB-DPs than control MB-DPs (Figure 4C). Principle component analysis and average distance analysis showed that the expression profile of MB-DP cells was similar to that of embryonic stem cells, whereas the gene expression profile of celecoxib-treated MB-DP cells were similar to MB-DN cells. (Figure 4D). Taken together, these results indicated that celecoxib treatment drove MB-DPs to lose their cancer stem-like gene signatures and differentiated into MB-DNs.

### 2.5. Celecoxib Enhanced the Sensitivity of MB-DP Cells to Radiotherapy

**Figure 5:**
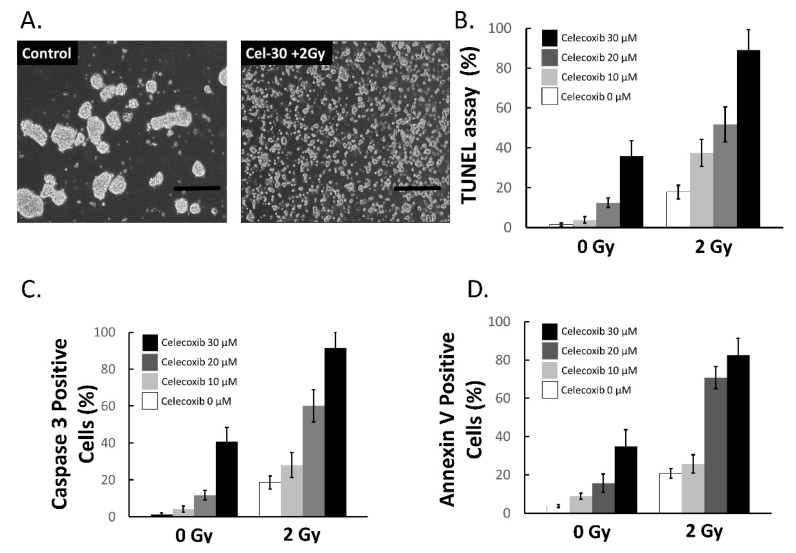
Celecoxib enhanced ionizing radiation-induced apoptosis in MB-DPs. (**A**) MB-DP cells (left) lose their sphere formation ability after treatment with celecoxib plus ionizing radiation (IR). Scale bar = 100 μm; and (**B**–**D**) Determination of TUNEL-positive cells, Caspase 3-positive cells and Annexin V-positive cells in MB-DP cells pretreated with various doses of celecoxib with or without following IR exposure (2 Gy). Data shown here are the mean ± SD of three experiments.

A recent study suggested that celecoxib could induce apoptosis of medulloblastoma cells including Daoy cells [36]. To determine whether celecoxib treatment could induce apoptosis in MB-DP cells and enhance the sensitivity of MB-DPs to ionizing radiation (IR), the changes of cell morphology and apoptosis marker expression levels were evaluated in celecoxib-treated MB-DP cells with or without IR (Figure 5A). Treatment of MB-DP cells with celecoxib plus IR decreased cell viability and the loss of sphere formation ability in MB-DP cells (Figure 5A). The results of MTT assay indicated that the LD_50_ of MB-DPs for celecoxib was found to be 30 μM (the data not show). Celecoxib treatment alone increased TUNEL-positive cells, Caspase 3-positive cells and Annexin V-positive cells in a dose-dependent manner, indicating the apoptosis of MB-DP cells induced by celecoxib (Figure 5B–D). Remarkably, exposure to 2 Gy IR alone induced moderate apoptosis in MB-DP cells, which was dose-dependently enhanced by celecoxib pretreatment (Figure 5B–D), revealing that celecoxib increased the sensitivity of MB-DP cells to radiotherapy. The efficacy enhancement of the combination was further confirmed by apoptosis assays. These data indicated that celecoxib could induce apoptosis and increased the sensitivity of MB-DP cells to radiotherapy.

### 2.6. Celecoxib and IR may Enhance the Inhibition on the Tumorigenicity of MB-DP Cells in Vivo

**Figure 6:**
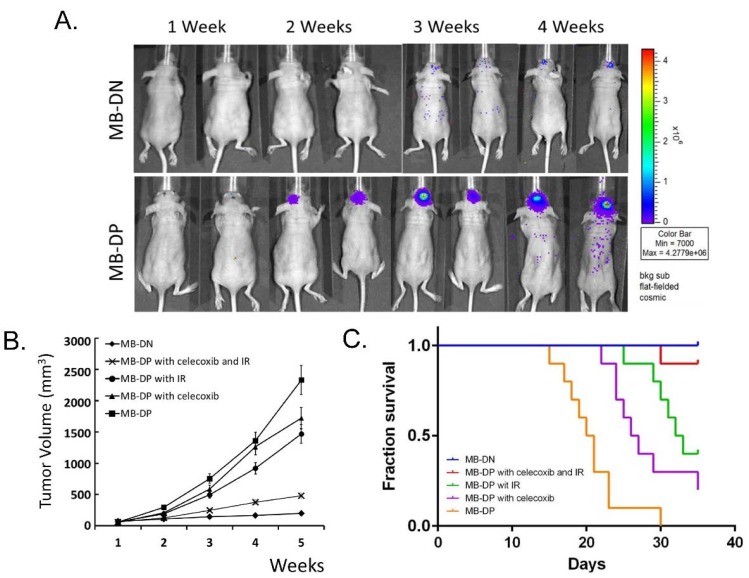
Celecoxib and irradiation may enhance the inhibition on MB tumorigenesis and recipient survival in orthotopic MB-DP-transplanted immunocompromised mice. (**A**) Representative bioluminescence images (BLI) of immunocompromised mice transplanted with MB-DP cells or MB-DN cells with firefly luciferase labeling at the indicated time points after cell inoculation; (**B**) Quantification of tumor volume in recipients of MB-DN cells or MB-DP cells treated with celecoxib, IR, or the combination of celecoxib and IR; and (**C**) The cumulative survival of MB-DP cells-xenotransplanted recipients receiving celecoxib, IR or the combination of celecoxib and IR. *p* < 0.05 *vs.* MB-DPs.

We further investigated the treatment effect of celecoxib that suppressed STAT3 phosphorylation and STAT3-related pathways on the *in vivo* tumorigenic ability of MB-DPs in orthotopic MB-DP-transplanted immunocompromised mice. Whether celecoxib showed a synergistic effect when used in combination with radiotherapy was simultaneously examined. During of the animal experiments, no side effects were observed in animals treated with celecoxib ± IR. As monitored by bioluminescence imaging (BLI), severe tumor formation was observed in all of the recipients of MB-DPs, but not MB-DNs (Figure 6A). IR exposure (4 Gy) or celecoxib administration alone moderately reduced tumor volume in recipients of MB-DP cells (Figure 6B). Importantly, the combination of celecoxib and IR exposure (4 Gy) largely reduced tumor volume in MB-DP tumor-bearing mice, indicating that celecoxib and IR synergistically reduced the tumor growth capability of MB-DPs (Figure 6B). Furthermore, either IR or celecoxib alone mildly improved recipient survival whereas the combination of IR (4 Gy) and celecoxib significantly prolonged the survival of MB-DP recipient, as compared with MB-DP recipients and MB-DP recipients with other treatment (Figure 6C). Taken together, these findings demonstrated that celecoxib is capable of suppressing MB tumorigenesis *in vivo*, and the combination of celecoxib and radiotherapy may be an effective strategy for the treatment of malignant MB tumorigenesis.

### 2.7. Discussion

Medulloblastoma (MB), which arises from cerebellum, is the most common brain malignancy tumor in childhood [37,38,39]. The cancer stem cell (CSCs) hypothesis has been proposed and may explain tumor aggressiveness and treatment failure in various malignancies. A rare population of tumor cells with cancer stem-like properties, known as cancer stem-like cells (CSCs) or cancer initiating cells, are able to undergo self-renewal and initiate tumors and may be responsible for tumor growth, resistance, and recurrence despite the application of conventional therapy [10]. Given that CSCs may serve as key contributors to the failure of conventional anticancer therapy, the therapeutic strategies targeting CSCs and CSC-associated mechanisms have drawn increasing attentions and are expected to improve the treatment of human cancers such as medulloblastoma.

STAT3 activation has been extensively demonstrated to serve a critical role in tumorigenesis in various tumors. For example, constitutive activation of STAT3 is the characteristics of various cancers and cancer cell lines [23,25,40]. Critical gene expression changes induced by aberrant activation of STAT3 may promote cancer cell growth, survival and inhibit apoptosis. [41,42,43]. In addition, another study reported that STAT3 may also have a role that promotes cancer cell invasion and metastasis [44]. Intriguingly, increasing evidence has shown that the inhibition of activated STAT3 may contribute to cancer cell death and tumor regression [12], and the inhibition of STAT3 could suppress the growth of cancer cells by decreasing cell proliferation and enhancing the apoptosis of cancer cells [41,45]. In this study, our findings have demonstrated that MB-DP cells with highly upregulation of STAT3-related genes also exhibited remarkable cancer stem-like signature, implying that this STAT3-related pathway may serve critical roles that may contribute to tumor aggressiveness in malignant MB.

Celecoxib was a COX-2-selective NSAID with anti-cancer activities that were exerted through the suppression of STAT3 phosphorylation [32]. Considering the inhibitory effect of celecoxib that specifically suppresses STAT3 activation, we examined the effect of celecoxib on cancer stem-like gene signatures, apoptotic cell numbers, and both the *in vitro* and *in vivo* tumorigenic properties. Interestingly, inhibition of STAT3 pathway by this drug shifted the gene signature of MB-DP cells to that similar to MB-DN cells (Figure 4). In addition, it also contributed to apoptosis of MB-DP cells (Figure 5) and largely suppressed their tumorigenic potential and sensitized the responsiveness of these MB-DPs to irradiation exposure *in vitro* (Figure 5) and in orthotopic MB-DP-transplanted recipients (Figure 6). Collectively, these observations of celecoxib effect highlighted the significance of STAT3-dependent pathway in MB, which is similar to previous reports addressing the role in STAT3 in other brain tumors [25,26]. STAT3-related pathways inhibition by celecoxib might possibly be one of the mechanisms of radiosensitization. The experimental results demonstrated that STAT3 pathways maybe not the only mechanisms to decrease stemness and increase radiosensitivity. In addition, since there have been several important reports on the mechanism of radiosensitization of tumor cells by celecoxib such as DNA repair inhibition [46,47,48], or endoplasmic reticulum (ER) stress loading [49]. Nevertheless, whether this celecoxib efficacy could be extended for clinical use of MB patient still requires further studies.

In the present study our findings have demonstrated that, at the molecular levels, celecoxib treatment largely downregulated the expression levels of phosphorylated-STAT3 and other STAT3-related genes in MB-DPs, shifted the gene signature of MB-DPs to a non-cancer stem-like pattern which exhibited limited tumorigenic potential. Celecoxib per se also restricted MB-DP growth and induced their apoptosis. Meanwhile, this drug showed a remarkable synergistic efficacy with irradiation exposure that reduced tumorigenicity in MB-DP cells and in orthotopically transplanted tumor *in vivo*. Taken together, the anticancer effects of celecoxib in medulloblastoma may be partly achieved through the STAT3 pathway.

## 3. Experimental Section

### 3.1. Flow Cytometry

The cell cycle distribution was analyzed using flow cytometry (BD FACSCalibur flow cytometer, BD BioSciences, San Jose, CA, USA) and BD CellQuest Pro software (version 5.1, BD BioSciences, San Jose, CA, USA) as described of manufacturer’s instructions. Cells were dissociated, antibody-labeled and resuspended in Hank’s balanced salt solution/2% fetal bovine serum. Experiments were repeated twice with at least three replicates.

### 3.2. Sphere and Soft Agar Assay

To analyze the sphere culture, Daoy after sorting for CD133 and Nestin were according to the protocol of Tirino *et al.* [29]. Briefly, after sorting for the CD133 and Nestin marker, sorted Daoy cells were plated at a density of 6 × 104 cells/well in 6-well ultra-low-attachment plates (Corning, Corning, NY, USA) in DMEM/F12 cell medium with human EGF (10 ng/mL), and human bFGF (10 ng/mL; Sigma, St. Louis, MO, USA). Fresh aliquots of epidermal growth factor (EGF) and basic fibroblast growth factor (bFGF) were added every day. After culture for 5~10 days, spheres were visible with an inverted phase-contrast microscope.

For the soft agar assay, the bottom of each well (35 mm) of a six-well culture dish was coated with 2 mL of an agar mixture (DMEM, 10% (*v*/*v*) FCS, 0.6% (*w*/*v*) agar). After the bottom layer solidified, 2 mL of a top agar-medium mixture (DMEM, 10% (*v*/*v*) FCS, 0.3% (*w*/*v*) agar) containing 2 × 10^4^ cells was added and incubated at 37 °C for 4 weeks. The plates were stained with 0.5 mL of 0.005% crystal violet, and the number of colonies was counted using a dissecting microscope.

### 3.3. Microarray Bioinformatics Analysis

Total RNA, cRNA probe preparation, array hybridization and data analysis were done as described of affymatriex’s manufacturer’s instructions. The AffymetrixTM HG-U133 Plus 2.0 whole genome chips were used (Santa Clara, CA, USA). RNA log expression units were calculated from Affymetrix GeneChip array data using the GeneSpring GX (Agilent, Santa Clara, CA, USA). The default settings were used to background correct, normalize and summarize all expression values. Significant difference between sample groups was identified using the *t*-test was calculated as normal for each gene and a *p*-value then calculated using a modified permutation test. Heatmap and Principle component analysis (PCA) was performed also by the GeneSpring GX software (Agilent, Santa Clara, CA, USA) to provide a visual impression of how the various sample groups are related. Gene annotation and gene Ontology were performed by the Amigo (http://amigo.geneontology.org/cgi-bin/amigo/go.cgi/).

### 3.4. RT-PCR

Total RNA was extracted using TRIzol reagent (Invitrogen, Carlsbad, CA, USA), according to the manufacturer’s protocol. RNA concentration and purity were determined by A260 and A260/A280 ratios, respectively. The integrity of total RNA was assessed on standard 1% agarose/formaldehyde gels. The RNA samples were treated with DNase I to remove residual traces of DNA. cDNA was obtained from 1 μg of total RNA, using reverse transcriptase (Promega, Madison, WI, USA) and random primers (Promega) in a final volume of 20 μL. cDNAs (1 μL/sample) were detected by PCR using “The Human JAK/STAT Signaling Pathway RT^2^ Profiler PCR Array”(QIAGEN, Valencia, CA, USA).

### 3.5. Immunoblotting

For western assays, the cells were treated with celecoxib or irradiation and homogenized in ice-cold buffer containing 250 mM sucrose, 10 mM Tris-HCl (pH 7.4), and protease inhibitors (1 mg/mL leupeptin, 1 mg/mL pepstatin, and 1 mM phenylmethylsulfonyl fluoride). These samples were centrifuged at 16,708× *g* for 10 min, and the supernatant was assayed for protein content using the SDS-PAGE assay (BIO-RAD Laboratories, Hercules, CA, USA). For SDS-PAGE, protein samples (20 mg/lane) were solubilized in Laemmli buffer, boiled at 90 °C for 5 min, subjected to electrophoresis on a 10% polyacrylamide gel, blotted to a polyvinylidene fluoride (PVDF) membrane (Millipore, Billerica, MA, USA), and immunoblotted using a primary antibody against the target protein. The specific proteins of interest were visualized using the enhanced chemiluminescence (ECL) detection system (GE Healthcare Life Science, Pittsburgh, PA, USA). Densitometric quantification of the bands was performed using the AlphaEaseFC software tool (Alpha Innotech, San Leandro, CA, USA). Western blotting was performed using standard protocols. The antibodies used for western were as follows: mouse monoclonal anti-Cox2 (Abcam, Cambridge, MA, USA), mouse monoclonal anti-Nestin (Abcam), rat monoclonal anti-PROM1 (Millipore), mouse monoclonal anti-JAK2 (Sigma), mouse monoclonal anti-BCL2 (BD Transduction, San Jose, CA, USA), mouse monoclonal anti-phosphotyrosine STAT3 (Cell Signaling, Danvers, MA, USA), mouse monoclonal c-Myc (BD Transduction). Blots probed with a mouse monoclonal anti-β-actin antibody (Clone AC-15, Sigma, St. Louis, MO, USA) were used to normalize the protein load. Stained bands were scanned, and intensity was quantified using the ImageJ (US NIH, Frederick, MD, USA). The experiment was performed in triplicate and repeated at least three times.

### 3.6. Apoptosis Assay

Cells were treated with increasing doses of celecoxib (0, 10, 20, 30 μM) with or without IR (2 Gy) for 48 h and then harvested and washed with phosphate buffered saline (PBS). After washing, the cells were stained with Annexin V-fluorescein isothiocyanate (FITC, BD Bioscience, San Jose, CA, USA) and propidium iodide (PI) for 15 min at 4 °C in the dark, in accordance with the manufacturer’s instructions (BD Biosciences, San Jose, CA, USA). After incubation, the cells were immediately analyzed using flow cytometry (EPICS XL, Beckman Coulter Inc., Fullerton, CA, USA). Early apoptotic cells stained positive for Annexin V-FITC and negative for PI. Late apoptotic cells were positive for both Annexin V-FITC and PI. The experiment was performed in triplicate and repeated at least three times. Furthermore, apoptotic cells were also identified by TUNEL assay (*In Situ* Cell Death Detection Kit, POD, Roche Applied Science, Indianapolis, IN, USA) and Caspase 3 were performed according to the manufacturer’s protocol.

### 3.7. In Vivo Analysis of Tumor Growth and Survival

All procedures involving animals were performed in accordance with the institutional animal welfare guidelines of Taichung Veterans General Hospital. For investigated the radiosensitizing effects of the celecoxib in treating medulloblastoma cell line by evaluating the growth of treated and untreated medulloblastoma cell line in 50 mice were according to the protocol of Ma *et al.* [34]. Briefly, the 6–8 weeks of age mice were obtained from the National Laboratory Animal Center (Taipei, Taiwan). They were divided into 5 groups (10 mice per group) were injected with each 5 categories of cells: MB-DNs, MB-DP treated with celecoxib (30 μM) and IR (2 Gy), MB-DP with IR only, MB-DP with celecoxib only and MB-DPs. The cerebellum of 6–8-week-old athymic nude mice were inoculated with 5 × 10^4^ each categories cells. Tumor size of each mouse was measured per 1 week by using *in vivo* bioluminescence imaging (BLI). Luciferase expression in MB cell lines was established by transfecting pGL4 luciferase expression vector (Promega) with neomycin resistance gene. To establish luciferase expression stable in MB cell lines, MB cell lines at 30% confluence were transfected by liposome-based gene delivery method. The procedures of Lipofectamine 2000 (Invitrogen, Carlsbad, CA, USA) and pGL4/Neo+ forming liposome/DNA complexes were according to manufacturer’s suggestions. In brief, 3 mg of pGL4/Neo+ were used to transfect MB cell lines cultured in 10 cm culture plate. Forty-eight hours after transfection, culture medium was replaced with neomycin (1 mg/mL; Sigma-Aldrich, St. Louis, MO, USA) containing culture medium for 3 weeks. Neomycin containing culture medium was replaced in every 3 days. Individual colonies of MB cell lines were picked up and the luciferase activities were determined by luciferase activities assay. MB cell lines with highest luciferase activities were selected and used for *in vivo* animal experiment. Mice were anesthetized with isoflurane (Forawick Vaporizer, Muraco Medical Co., Tokyo, Japan). Seven minutes after intraperitoneal injection with 75 mg/kg body weight *n*-Luciferin (Xenogen Corp., Alameda, CA, USA), the mice were imaged in a Xenogen IVIS50 imaging system (Xenogen Corp., Alameda, CA, USA) coupled to a charge-coupled device (CCD) camera. After injection of the *n*-Luciferin, images were acquired for 2 min with the Xenogen IVIS system (Xenogen, Alameda, CA, USA) using LivingImag™ software and acquisition software (Xenogen). Regions of interest were drawn around discrete anatomical areas and used to calculate bioluminescent signals. This signal was expressed and graphed as total flux (photos/second). All injected mice were routinely screened with BLI, and image-positive mice were followed over time, treated and followed over time. Animals were killed at 10 weeks after cell injection, or earlier if they showed evidence of neurological symptoms related to increased intracranial pressure or if tumor volume exceeded 2500 mm^3^ as estimated by BLI measurement.

### 3.8. Survival

Another 50 mice (5 groups of 10) were injected with 5 × 10^4^ medulloblastoma cell line from the same 5 categories as described above. Animals were killed when they showed evidence of neurological symptoms due to increased intracranial pressure or when tumor volume exceeded 2500 mm^3^, as estimated by BLI imaging measurement, in order to obtain brain specimens were examined for verification of tumor-related influence on survival. Any remaining mice were killed at 10 weeks after cell injection.

### 3.9. Statistical Analysis

Statistical analysis was performed using SPSS software (SPSS, Inc., Chicago, IL, USA) to conduct the statistical analysis of the data. For comparisons between two samples, an unpaired two-tailed *t*-test was performed. A *p*-value of <0.05 was considered to be statistically significant. The results are reported as means ± SDs. Survival and time to progression were estimated by the Kaplan–Meier method and compared by log-rank analysis.

## 4. Conclusions

Celecoxib significantly reduced the expression level of phosphorylated signal transducer and activator of transcription 3 (p-STAT3) and cancer stem-like cell abilities. These results suggested a crucial role of STAT3-related pathway in the tumor progression in tumors arised from MB-DPs. The combination of celecoxib and radiotherapy is a promising strategy to overcoming resistance of medulloblastoma patients. Our results indicated that celecoxib may be a radiosensitizing drug for clinical use in patients with medulloblastoma.
